# Immobilization of Peroxidase on Functionalized MWCNTs-Buckypaper/Polyvinyl alcohol Nanocomposite Membrane

**DOI:** 10.1038/s41598-019-39621-4

**Published:** 2019-02-18

**Authors:** Lau Yien Jun, N. M. Mubarak, Lau Sie Yon, Chua Han Bing, Mohammad Khalid, Priyanka Jagadish, E. C. Abdullah

**Affiliations:** 1 Department of Chemical Engineering, Faculty of Engineering and Science, Curtin University, 98009 Sarawak, Malaysia; 2 Graphene & Advanced 2D Materials Research Group (GAMRG), School of Science and Technology, Sunway University, No. 5, Jalan University, Bandar Sunway, 47500 Subang Jaya, Selangor Malaysia; 3 Department of Chemical Process Engineering, Malaysia-Japan International Institute of Technology (MJIIT) Universiti Teknologi Malaysia (UTM), Jalan Sultan Yahya Petra, 54100 Kuala Lumpur, Malaysia

## Abstract

Surface modified Multi-walled carbon nanotubes (MWCNTs) Buckypaper/Polyvinyl Alcohol (BP/PVA) composite membrane was synthesized and utilized as support material for immobilization of Jicama peroxidase (JP). JP was successfully immobilized on the BP/PVA membrane via covalent bonding by using glutaraldehyde. The immobilization efficiency was optimized using response surface methodology (RSM) with the face-centered central composite design (FCCCD) model. The optimum enzyme immobilization efficiency was achieved at pH 6, with initial enzyme loading of 0.13 U/mL and immobilization time of 130 min. The results of BP/PVA membrane showed excellent performance in immobilization of JP with high enzyme loading of 217 mg/g and immobilization efficiency of 81.74%. The immobilized system exhibited significantly improved operational stability under various parameters, such as pH, temperature, thermal and storage stabilities when compared with free enzyme. The effective binding of peroxidase on the surface of the BP/PVA membrane was evaluated and confirmed by Field emission scanning electron microscopy (FESEM) coupled with Energy Dispersive X-Ray Spectroscopy (EDX), Fourier transform infrared spectroscopy (FTIR) and Thermogravimetric Analysis (TGA). This work reports the characterization results and performances of the surface modified BP/PVA membrane for peroxidase immobilization. The superior properties of JP-immobilized BP/PVA membrane make it promising new-generation nanomaterials for industrial applications.

## Introduction

The current trend of green and sustainable technologies has encouraged the implementation of enzymatic technology in industrial processes due to its simplicity, efficiency, economical and eco-friendliness^1^. Peroxidases (E.C. 1.11.1.7) are oxidoreductases which are a widely studied enzyme group that mostly functions as a catalyst in the oxidation of a broad variety of organic compounds in the presence of hydrogen peroxide^2^. Numerous studies devoted to applications of peroxidases, particularly plant peroxidases, in the area of biotechnology, biomedical, wastewater treatment, and food processing have been reported earlier^3,4^. Plant peroxidases can easily be extracted from various types of plants, such as horseradish, soybean, turnip, ginger, bitter gourd, and jicama^5,6^. Despite the peroxidase advantages, its practical application at large scale is still restricted due to some of its undesirable properties. For instance, the high sensitivity of catalytic activity to environmental conditions, low storage operational stabilities, and the lack of enzyme recovery and reusability^7–9^. To overcome these limitations, numerous studies on immobilization of peroxidases on different support materials have been performed^10–12^.

Selection of appropriate immobilization method, support materials, and operating conditions for enzyme immobilization plays a critical role in achieving superior enzyme performances and stabilization. Generally, the immobilization methods are categorized into physical and chemical methods, which are adsorption, covalent bonding, cross-linking, entrapment and encapsulation^13–15^. The existing support materials can be divided into organic and inorganic with respect to their chemical compositions. The most commonly used support materials are silica, clay, kaolin, glass bead, chitosan, and polystyrene^16,17^. Nowadays, the success of nanostructured support materials, such as nanoparticles, nanotubes, and nanocomposites, have received increasing attention from various researchers due to their desirable properties^18–20^. These nanostructured materials have a large surface area to volume ratio, immensely porous and hollow structures, high thermal and mechanical properties^21,22^. Moreover, the surface properties of the nanostructured support materials can be engineered and tailored according to the application required^23^. Despite the superior properties of nano-structured support materials for enzyme immobilization technologies, it is impractical to use nano-scale materials for scaling-up and industrial continuous operating systems^24^. Their extremely small sizes resulted in difficult for separation from the reaction medium for further recovery and reusability purposes.

The application of buckypaper/polyvinyl alcohol (BP/PVA) nanocomposite membrane as the support material for immobilization of peroxidase has overcome the separation problem. A freestanding BP-reinforced PVA was synthesized from functionalized multi-walled carbon nanotubes (f-MWNCTs) into a macroscopic sheet, followed by filtering the membrane formed with PVA solution using vacuum infiltration method. Strong interfacial interaction between BP and polymer matrices is vital for improving the physical properties and performances of the nanocomposite membrane. Thus, the selection of an appropriate polymer is one of the significant factors. In this study, PVA was chosen as the reinforcement polymer since the hydroxyl groups in PVA can form strong hydrogen bonding with the hydrophilic surface of f-MWCNTs. Besides, previous studies show BP/PVA membrane as a promising next-generation nanocomposite material due to its robust thermal, mechanical, electrical and electromechanical properties^25,26^. The outstanding physicochemical properties of BP/PVA membrane make them promising candidates for extensive future application in various fields, such as energy conservation, biotechnology, environmental and biomedical^27,28^. Jicama peroxidase (JP) was chosen in this study as the enzyme owing to its high selectivity, high substrate specificity, polyfunctionality and its availability from agricultural wastes^29^. Compared to conventional enzyme immobilization technologies, the integration of enzyme immobilization with nanocomposite membrane system could offer more advantages. For instance, higher enzyme loading capabilities, improved operational stabilities and enzymatic efficiencies, prolonged membrane lifetime and, ease of separation, effective regeneration and reusability of bio-adsorbent^30,31^. In addition, it can reduce operating costs by reducing the enzyme required due to its long lifespan and enhanced reusability^32,33^.

To the best of the authors’ knowledge, the study of using BP/PVA nanocomposite membrane as a support matrix for enzyme immobilization has yet to be reported. Thus, the main objective is to study the immobilization of peroxidase extracted from jicama skin peels on functionalized BP/PVA membrane via covalent bonding. Besides, another aim is to develop a robust and reliable optimization model for enzyme immobilization efficiency on BP/PVA membrane. The optimization of enzyme immobilization parameters was performed by using Response surface methodology (RSM). RSM is used in this work by adopting the face-centered central composite design (FCCCD). The developed optimization framework also served as a platform for further study of other parameters, such as pH, thermal and storage stabilities. Both operational stabilities of free and immobilized enzymes were compared and analyzed. Characterization studies of JP-immobilized BP/PVA membrane were performed using as FESEM, EDX, FTIR, and TGA to facilitate in evaluating the biocompatibility of the peroxidases and BP/PVA membrane. Overall, this study provides an insight on the mechanism of enzyme immobilization on a nano-structured support material.

## Results and Discussion

### Activity and Protein Concentration of Free Enzyme

**Figure 1:**
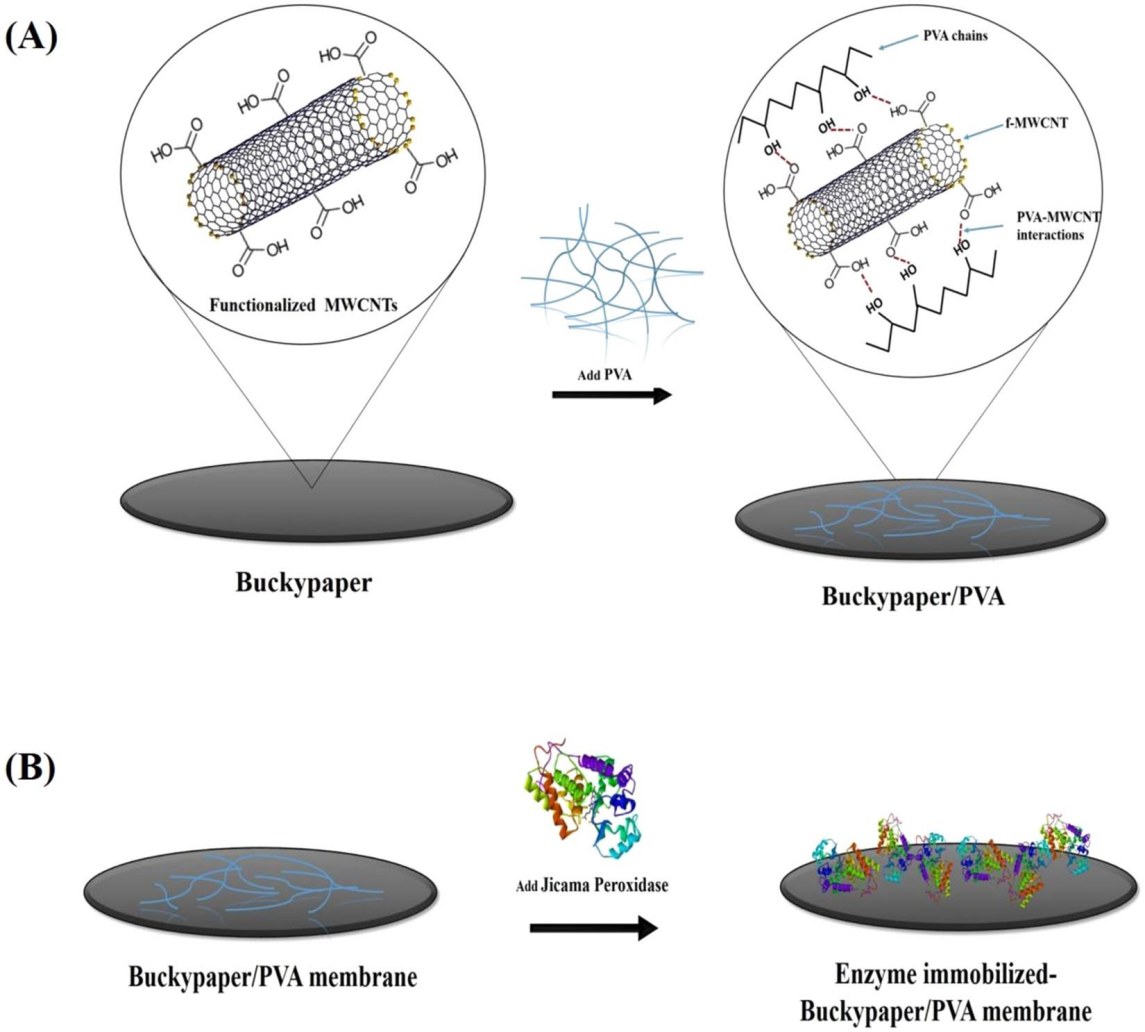
Schematic Diagram of (**A**) Synthesis of BP/PVA membrane; (**B**) Synthesis of Enzyme immobilized-BP/PVA membrane.

Based on the experimental results, the free enzyme has an average activity of 1.40 U/mL, protein concentration of 2.92 mg/mL and specific activity of 0.48 U/mg. Similar results were reported by Chiong, *et al*.^34^, where 1.22 U/mL JP was extracted from the jicama skin peels. The free enzyme was then covalently bonded to BP/PVA membrane support by using GA as the crosslinking agent. Figure 1 shows the schematic diagram of the synthesis of BP/PVA membrane and the subsequent immobilization of JP on the membrane.

### Enzyme Immobilization using Regression Models and Statistical Testing

In order to obtain the optimum enzyme immobilization efficiency, RSM was used. The obtained experimental immobilization efficiency was analyzed to develop a suitable regression model.

A second order quadratic regression surface model was fitted to the experimental results to obtain the predicted result of the enzyme immobilization efficiency. The relationship between the experimental variables and response are fitted to a quadratic polynomial equation as shown in Eq. (1):

1 $$\begin{array}{rcl}Y & = & +{\rm{72.73}}-2.13\,A-6.47\,B+1.44\,C\\  &  & +\,1.42\,AB+0.0314\,AC-0.2104\,BC\\  &  & -\,8.05\,{A}^{2}+3.45\,{B}^{2}-9.31\,{C}^{2}\end{array}$$

where the immobilization efficiency (Y) is a function of pH (A), initial enzyme loading (B) and immobilization time (C) respectively.

Analysis of variance (ANOVA) was performed to determine the significance of the model by checking several criteria, such as F-values, p-values, lack of fit test and regression coefficient values. P-values lower than 0.05 indicate that model terms are significant. A high model F-value of 72.19 and a low model p-value of <0.0001 indicated that there is only a 0.01% probability that a model with F-value this large could happen owing to noise and these values indicate that the model is significant. The model also depicted a non-significant lack of fit behavior with a p-value of 77.69% which shows that the experimental data fits well with the model used.

Additionally, the determination coefficient, R^2^ was evaluated as 0.9909, and the adjusted R^2^ was 0.9771. Both values were closed to 1, which displayed a relatively high degree of correlation between the actual and predicted responses. The predicted R^2^ of 0.9556 was in reasonable agreement with the adjusted R^2^, as the difference is less than 0.2. Generally, a ratio larger than 4 is preferred for the precise signal to noise ratio in a model. In this study, the ratio was found to be 30.47, which indicated a good signal to noise ratio balance which is sufficient to maneuver through the design space. The results of the ANOVA is as depicted in Table 1.

**Table 1:** ANOVA for RSM.

Source	Sum of Squares	df	Mean Square	F-value	p-value	
Model	1167.30	9	129.70	72.19	<0.0001	significant
A - pH	45.54	1	45.54	25.35	0.0024	
B - Initial Enzyme Loading	418.54	1	418.54	232.96	<0.0001	
C - Immobilization Time	20.87	1	20.87	11.61	0.0144	
AB	16.19	1	16.19	9.01	0.0240	
AC	0.0079	1	0.0079	0.0044	0.9493	
BC	0.3542	1	0.3542	0.1971	0.6726	
A²	171.01	1	171.01	95.19	<0.0001	
B²	31.46	1	31.46	17.51	0.0058	
C²	228.67	1	228.67	127.28	<0.0001	
Residual	10.78	6	1.80			
Lack of Fit	7.77	5	1.55	0.5172	0.7769	insignificant
Pure error	3.01	1	3.01			
Cor total	1178.08	15				

**Figure 2:**
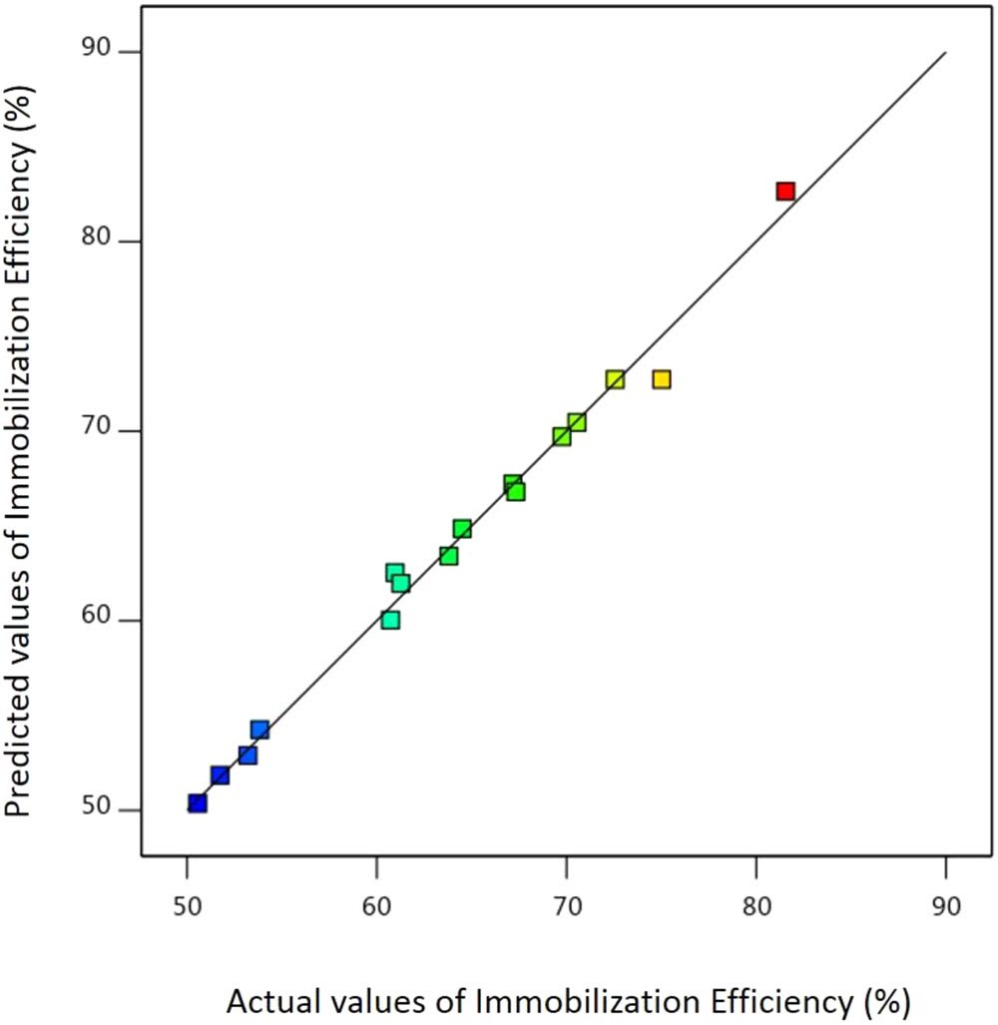
The plot of relationship between predicted and actual values of enzyme immobilization efficiency.

Figure 2 presents the predicted values versus actual values plot for enzyme immobilization efficiency. The result indicated that the results of model prediction were close to the actual experimental values. Thus, the developed model was proved to be a effective platform to bridge the correlation between process parameters to the enzyme immobilization efficiency^35,36^.

### Effect of Process Parameters on Enzyme Immobilization Efficiency

**Figure 3:**
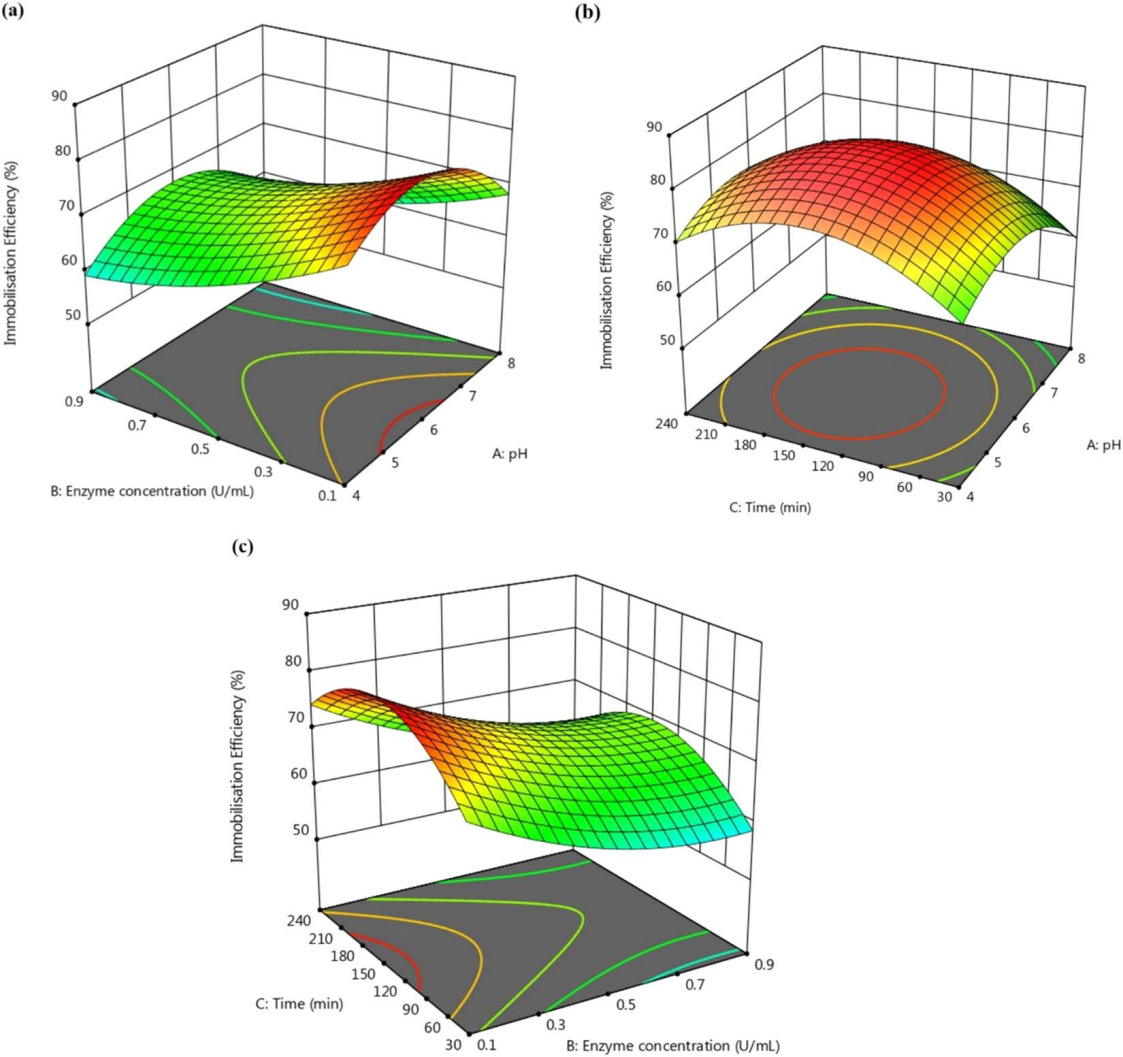
3D response surface plots for enzyme immobilization efficiency as a function of (**a**) Initial enzyme loading and pH; (**b**) Immobilization times and pH; (**c**) Immobilization times and initial enzyme loading.

The 3D response surface plots for the interaction between two independent variables on enzyme immobilization efficiency are presented in Fig. 3. Figure 3(a) demonstrates the response surface plot for the effects of pH and initial enzyme loading on peroxidase immobilization efficiency on BP/PVA membrane. It is evident that the pH had a notable impact on the enzyme immobilization efficiency. Figure 3(a) shows an increment in enzyme immobilization efficiency from 78% to 83% when the pH ranges were varied from 4 to 5 at constant initial enzyme loading of 0.1 U/mL and immobilization time of 135 min. However, a further increase in pH will lead to decrease in immobilization efficiency. This might be due to the change in the enzyme protein structures under these pH conditions^37^. Additionally, the results also revealed that the low initial enzyme loading of 0.1 U/mL was sufficient to cover all the pore sites on the surface of the BP/PVA membrane. Therefore, the immobilization efficiency started to decrease with further increase in the enzyme loading as it eventually reaches its saturation level. Figure 3(a,c) indicates that excess enzyme loading leads to a decrease in the enzyme immobilization efficiency. Hence, high enzyme loading was not economical for enzyme immobilization due to the loss of free enzymes as the excess enzymes were washed off during the immobilization process, resulting in higher filtration resistances^38^. According to Pramparo, *et al*.^39^, a higher amount of free enzyme loading caused a decrease in coupling yield as they were not bonded to the surface of the support materials.

Figure 3(b) denotes the 3D interaction plot between enzyme immobilization time and pH with the constant initial enzyme loading of 0.5 U/mL. As seen from the results in Fig. 3(b), the maximum enzyme immobilization efficiency of 82% was achieved when the interactions between both factors were at pH range of 5–6, and immobilization time of 120–150 min. Additionally, Fig. 3(c) depicts the 3D response surface for the enzyme immobilization efficiency as a function of immobilization time and initial enzyme loading. The enzyme immobilization efficiency increased from 72% to 81% as the immobilization time increased from 30 to 150 min. Nevertheless, the immobilization efficiency decreased as the time was prolonged beyond 150 min.

### Optimization of Operating Conditions for Immobilization Efficiency

Based on the ANOVA and 3D surface plot results, the optimum conditions for the enzyme immobilization efficiency were obtained at pH 6, 0.13 U/mL of initial enzyme loading for 130 min immobilization time, with maximum enzyme immobilization efficiency of 81.74%.

### Validation

From the optimized conditions, three replicates of the experiments were performed to verify the prediction. A comparison was made between the experimental data and model predictions for the enzyme immobilization efficiency. The average immobilization efficiency obtained was 81.03%, which was very close to the predicted value by the model (81.74%).

Table 2 summarizes the comparison of peroxidase immobilization efficiency and protein loading on their supports between previous studies and the current study. Notably, enzyme immobilization on nano-structured support materials can achieve higher enzyme loading and immobilization efficiency as compared to bulk materials. The use of surface modified BP/PVA membrane for JP immobilization can provide the highest enzyme loading compared to other existing support materials due to its large surface area to volume ratio, highly porous structure, as well as high oxygenated functional groups on its surface.

**Table 2:** Comparison of the Peroxidase Immobilization Efficiency and Protein loading.

Enzymes	Support materials	Immobilization Efficiency (%)	Protein content (mg/g dry support)	References
HRP	Kaolinite	35.5	1.7	^11^
HRP	Vermiculite	56.2	2.6	^11^
HRP	Montmorillonite	66.0	3.1	^11^
HRP	Magnetic poly(GMA-MMA)-GA beads	67.0	3.4	^72^
HRP	Porous aminopropyl glass beads	37.0	9.6	^73^
SBP	Porous glass	28.0	13.3	^74^
SBP	Alkylamine glass	45.2	34.9	^75^
HRP	Alkylamine glass	40.6	18.3	^75^
SBP	Aldehyde glass	50.9	42.3	^76^
HRP	Aldehyde glass	31.5	23.1	^76^
HRP	Chitosan Halloysite hybrid-nanotubes	—	21.5	^3^
HRP	Graphene oxide	—	100.0	^47^
HRP	Zinc oxide (ZnO) nanowires/macroporous silicon dioxide (SiO_2_)	75.3	161.3	^40^
HRP	Tin dioxide (SnO_2_) hollow nanotube	77.6	181.0	^77^
JP	BP/PVA	81.0	217.3	Present study

### Effect of pH on the Free and Immobilized Enzymes

**Figure 4:**
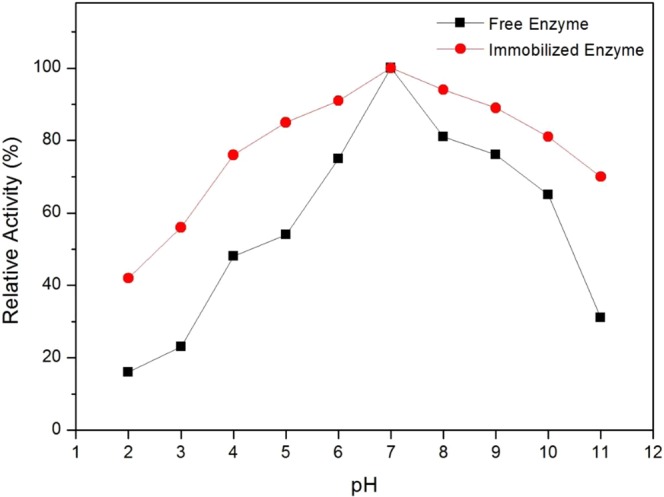
Effect of pH on the relative activity of (**a**) free enzyme and (**b**) immobilized enzyme at 30 °C.

The relative activity of free and immobilized JP on BP/PVA membrane as a result of varying pH is shown in Fig. 4. Both free and immobilized peroxidase showed the highest relative activity at pH 7. Besides, immobilized JP can retain more than 80% of its activity after incubation at pH conditions of 5 to 10. Conversely, free enzyme loses more than 50% of its enzymatic activity at pH lower than 5 and pH above 10. Overall, immobilized JP exhibited higher enzymatic activity over a wider range of pH compared to that of free enzyme. The increase in pH stability of immobilized JP is due to the strong covalent attachment of the enzyme molecules to the support matrix. The strong intermolecular forces cause the immobilized enzymes highly resistant towards the environmental changes. In addition, the stable binding with support material also prevents the conformational changes of enzymes under extreme pH conditions, and hence restricting enzyme denaturation^40,41^. Immobilized enzymes have better acidic and alkaline tolerance stability, making it useful for diverse applications. In agreement with our findings, the increase of pH stability following immobilization had formerly been observed by several researchers^40,42–44^.

### Effect of Temperature on the Free and Immobilized Enzymes

**Figure 5:**
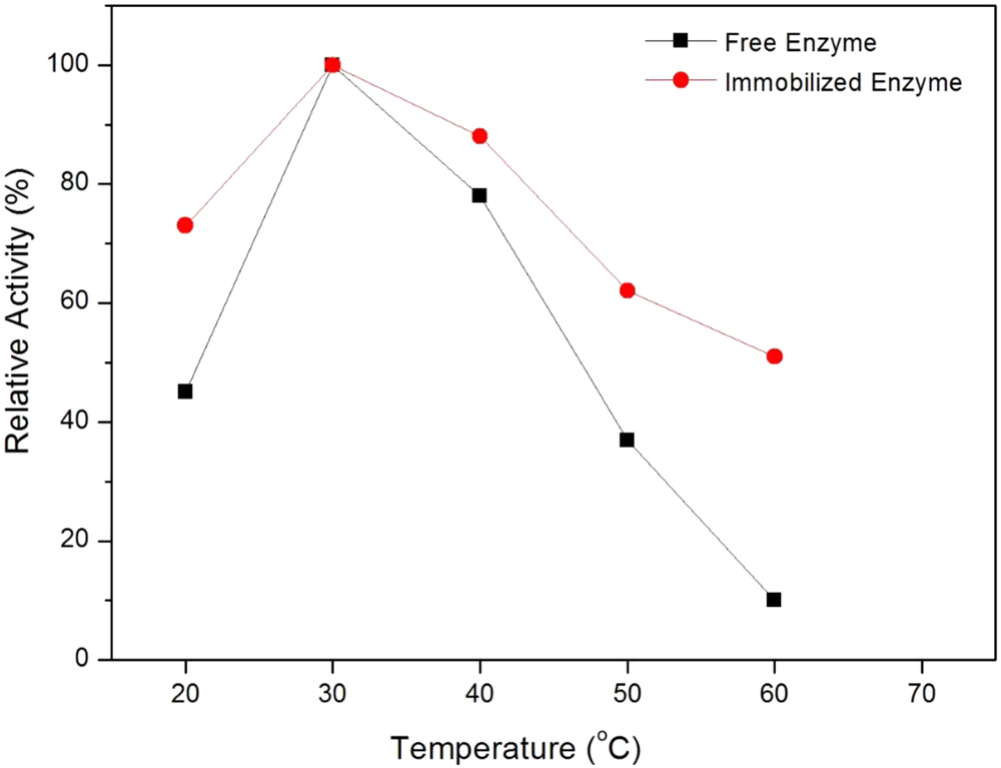
Effect of temperature on the relative activity of (**a**) free and (**b**) immobilized enzyme at pH 7.

Figure 5 depicts the effect of temperatures on the relative activities of free and immobilized enzymes. The temperature for optimum relative activities for both free and immobilized enzymes are at 30 °C. The immobilized peroxidase can retain more than 80% of its relative activity from 30 to 40 °C. In contrast, the relative activity of free enzyme declined drastically when the temperatures were above 40 °C. The loss in enzymatic activity of free enzymes at high temperatures was caused by structural denaturation of enzymes. The improved heat resistance of immobilized enzyme was due to its protection from conformational changes imposed by heat^40,44–46^.

**Figure 6:**
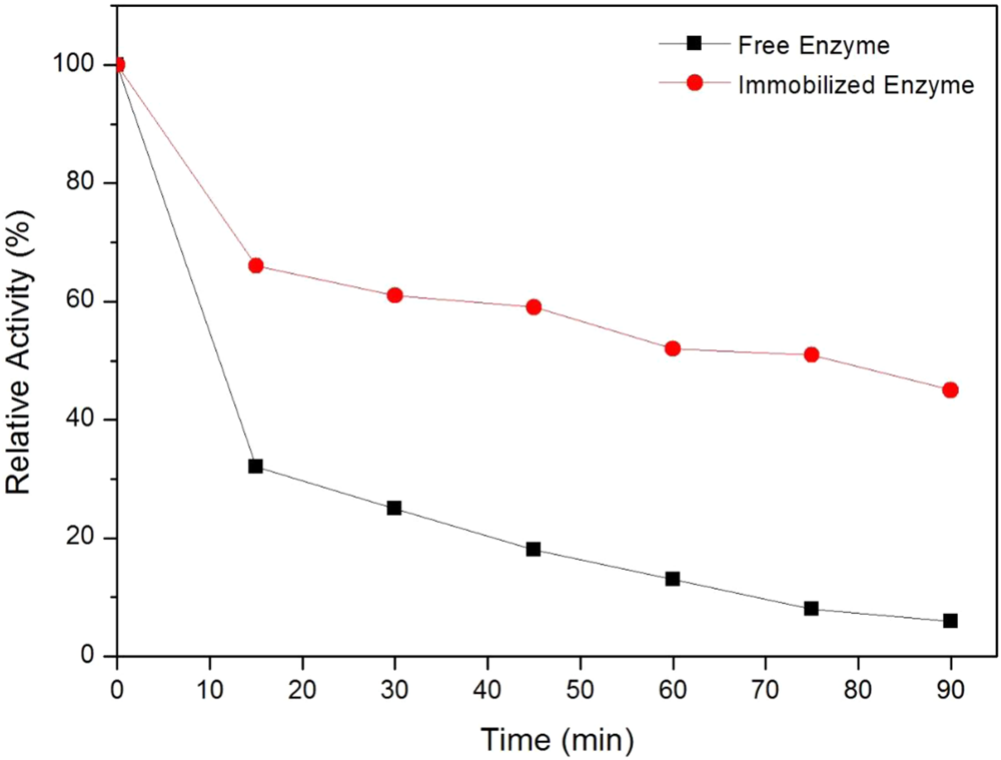
Thermal stabilities of (**a**) free and (**b**) immobilized enzyme at 50 °C for 90 min.

In addition, Fig. 6 shows the comparison of thermal stabilities of free and immobilized enzymes in term of the relative activities at 50 °C. Results showed that the immobilized JP retained 45% of its relative activities at 90 min, while free enzyme experienced a huge loss of its activity under similar operating parameters. Besides, previous studies also confirmed the enhancement of thermal stability of enzymes after immobilization^43^. The strong covalent interaction between the enzyme molecules to the BP/PVA membrane support improved the thermal stability of immobilized enzymes. The strong covalent bonding restricts the protein mobility at high temperatures, imparting rigidity to the enzyme structure and prevent the enzyme from denaturation^40^. Thus, these results showed that immobilization led to a significant effect on the thermal stability of the enzyme.

### Storage Stability of Free and Immobilized Enzyme

**Figure 7:**
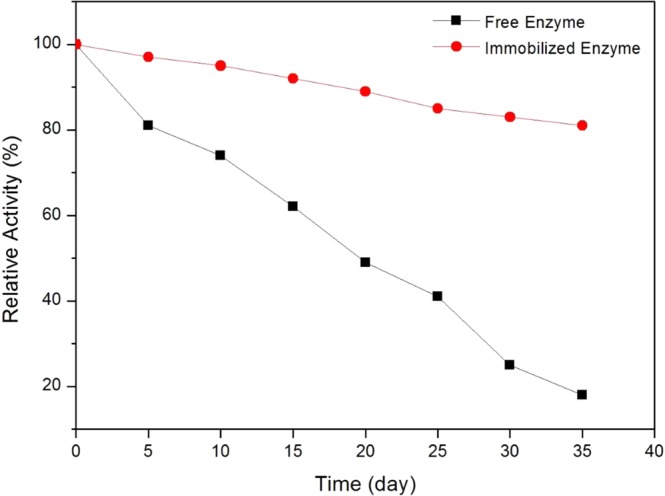
Storage stabilities of free and immobilized JP at 4 °C for 5 weeks.

Figure 7 presents the long-term stabilities of the free and immobilized enzyme activity at 4 °C for 5 weeks. After 35 days, the immobilized and free enzyme retained 81 and 18% of its initial activity respectively. Immobilized enzyme demonstrated longer storage lifetime as compared to free enzyme. This shows that the BP/PVA support could provide a stabilizing effect, due to the intra and inter-molecular cross-linking. Therefore, the immobilized enzyme can resist conformational changes and minimize distortion on the active sites of enzyme imposed from the aqueous medium^42,47^.

### Characterization studies of BP and Enzyme-Immobilized BP

#### FESEM morphology

**Figure 8:**
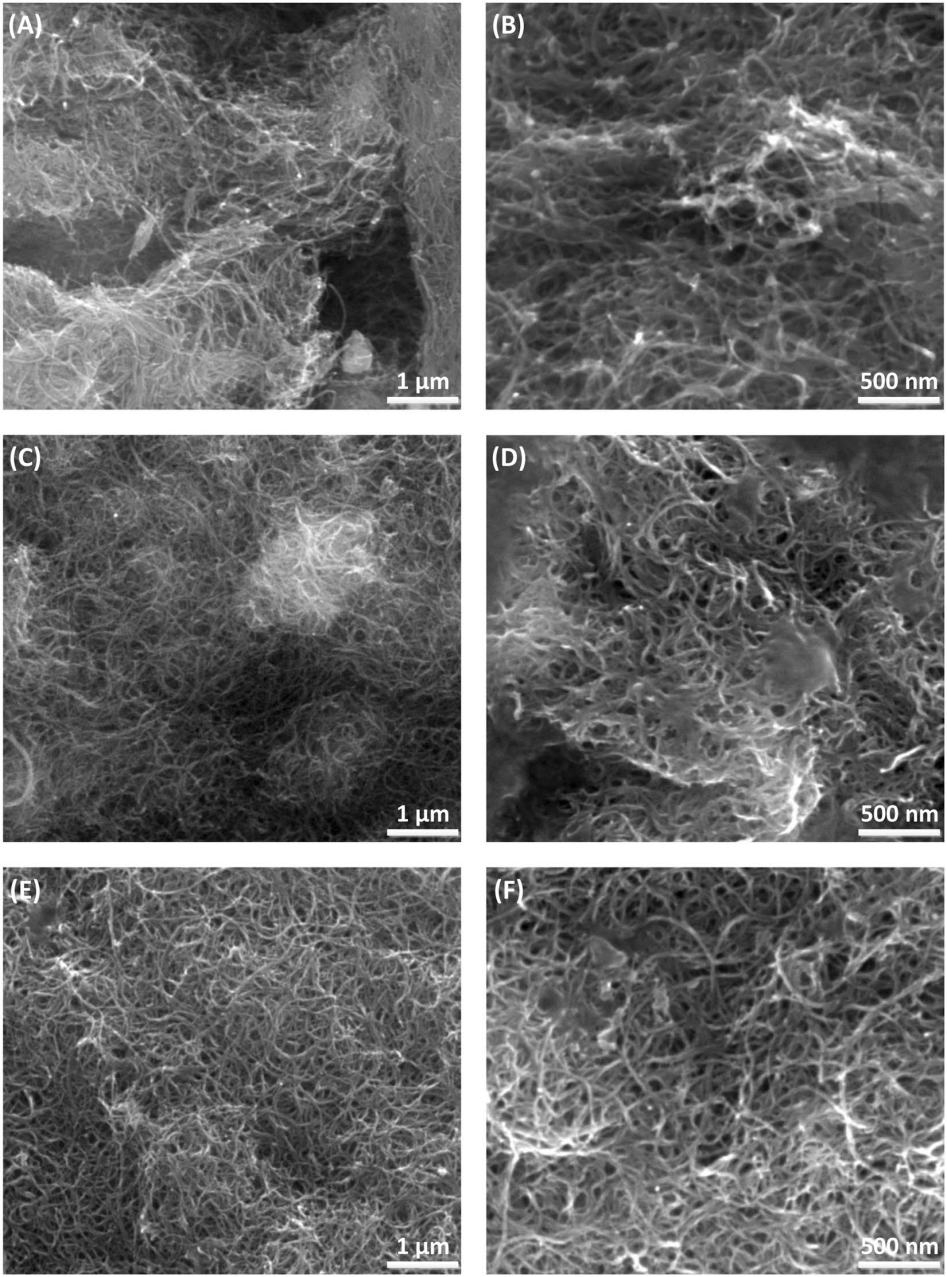
FESEM images of (**A**,**B**) BP, (**C**,**D**) BP/PVA and (**E**,**F**) JP-immobilized BP/PVA membranes under magnifications of 80,000 × (1 µm) and 120,000 × (500 nm) respectively.

To elucidate the effect of immobilization of peroxidase on the membrane, FESEM study was conducted to study the structural and surface morphologies of the membranes. FESEM images of the BP, BP/PVA and enzyme-immobilized BP/PVA membranes are depicted in Fig. 8. As seen in Fig. 8(A,B), a fibrous structure of the BP with entanglements of MWCNTs bundles can be observed. The images in Fig. 8(C,D) show the diameter of BP/PVA membrane was obviously thicker than the original BP membrane owing to the attachment of PVA polymer layer its surface. It was also observed that the surface of BP/PVA membrane was porous and smooth before enzyme immobilization was performed. As evidence of enzyme immobilization, Fig. 8(E,F) indicates JP-immobilized BP/PVA membrane having a rougher surface with more saturated pores. The highly porous membrane surface of the support can increase the density of enzyme loading capacity^48^. Besides, the occurrence of agglomeration was primarily due to the formation of covalent bonds between the carboxylic groups of BP/PVA membrane and the amine group of enzymes via glutaraldehyde as crosslinking agent^49^. The FESEM images confirmed the enzyme molecules were successfully attached on the surface of BP/PVA membrane.

#### EDX analysis

**Figure 9:**
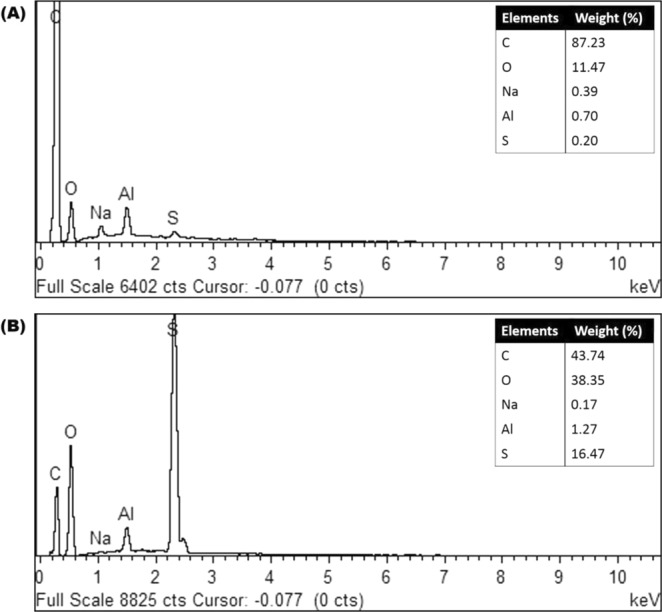
EDX analysis of (**A**) BP/PVA membrane and (**B**) JP-immobilized BP/PVA.

To determine the elements, present on the membranes surface, both BP/PVA and JP-immobilized BP/PVA membranes were analyzed by using EDX. The EDX results in Fig. 9 displays the presence of various elements in both the samples, which are carbon C, oxygen O, aluminum Al, sodium Na, and sulphur S. The result shows that JP-immobilized BP/PVA membrane exhibited a higher mass fraction of oxygen content (38.35 wt. %) as compared to the BP/PBA membrane (11.47 wt. %). Thus, the increased intensity of oxygen content confirmed the successful immobilization of JP on BP/PVA membrane. Similar behavior has been observed for immobilization of HRP onto iron magnetic nanocomposite^50^. Besides, Sahare, *et al*.^51^ also reported an increase in oxygen content for the immobilization of HRP onto mesoporous silicon/silica micro-particles.

#### FTIR analysis

**Figure 10:**
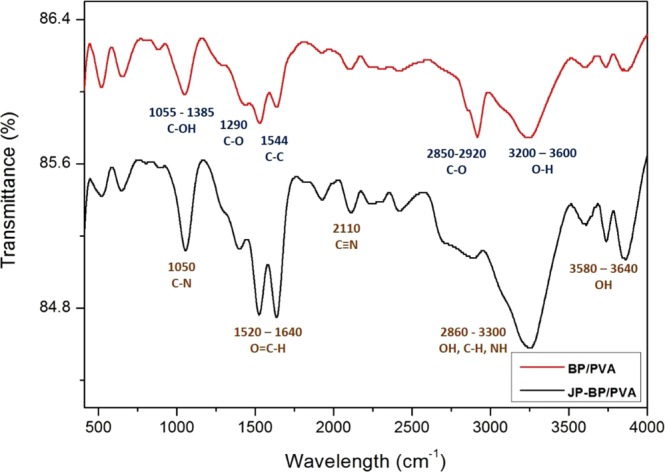
FTIR spectra of (**A**) BP/PVA membrane and (**B**) JP-immobilized BP/PVA.

In order to verify the immobilization of peroxidase on BP/PVA membrane, Fourier Transform Infrared Spectroscopy (FTIR) study with a wavelength range of 400–4000 cm^−1^ were performed. Comparison of FTIR spectrum of BP, BP/PVA membrane and enzyme-immobilized BP/PVA membrane is illustrated in Fig. 10. By comparing the spectrum of BP/PVA and JP-immobilized BP/PVA membranes, both samples spectra showed similar bands for several peaks. For instances, C-O stretch at 1190–1400 cm^−1^, C-C bond at 1544 cm^−1^, C=C aromatic bending at 1320–1550 cm^−1^, and C-H stretching bond at 2851–2919 cm^−1^ ^52^.

Besides, the intensive peaks at 1665–1740 cm^−1^, 1055–1385 cm^−1^ and 2500–3610 cm^−1^ were attributed to C=O, C-OH and O-H bonds respectively. The presence of these oxygenated functional groups, such as hydroxyl, epoxide and carboxyl groups indicated the effective surface functionalization of MWCNTs^53,54^. In addition, the wide peaks at 3200–3600 cm^−1^ indicated the presence of hydrogen-bonded hydroxyl groups of PVA in BP/PVA membrane^55^. Moreover, the peaks at 2840–2920 cm^−1^ correspond to the stretching C-O bond, which represents the CH_2_ groups of PVA^56^. This suggests that the structures of PVA and MWCNTs backbones were retained even after enzyme immobilization.

After the immobilization of JP, there were few peaks with increase intensities at 1020–1250 cm^−1^, 1520–1640 cm^−1^ and 2210–2260 cm^−1^, indicating the aliphatic amide bond (C-N), amide groups (O=C-NH), nitriles (C≡N) respectively^57,58^. The presence of these acylamino groups (combination of –CHO and –NH_2_) indicates amidination reaction and confirmed the immobilization of JP on the BP/PVA membrane^59^. This statement is further supported by the previously reported studies^60–62^. Additionally, the crosslinks between functionalized BP/PVA membrane and glutaraldehyde are detected with an increase in the intensity of C-H band at 2850–3270 cm^−1^ ^42^. Aldehyde groups were generated to the BP/PVA membrane through the reaction with glutaraldehyde. These aldehyde groups could react specifically with –OH groups on the surface of BP/PVA membrane and attached to the alpha-amino residues of enzymes^63^. Therefore, all of these results verified the successful immobilization of JP on the BP/PVA membrane support via covalent bonding.

### Thermogravimetric Analysis

**Figure 11:**
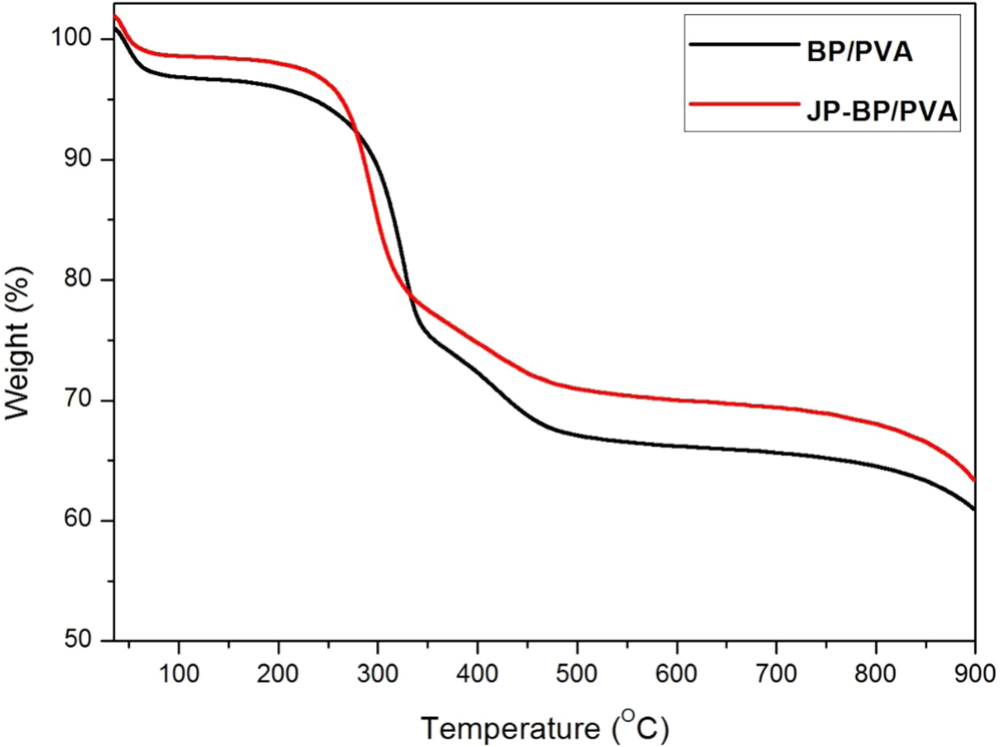
TGA curves of (**A**) BP/PVA membrane and (**B**) enzyme immobilized BP/PVA membrane.

The immobilization of JP on BP/PVA membrane was further investigated by TGA to determine crystallinity, thermal stability of the membranes, as well as their constituents. Figure 11 illustrates the TGA curves of both BP/PVA and JP-immobilized BP/PVA membranes.

The TGA weight loss curves for both BP/PVA and JP immobilized BP/PVA membranes exhibited several weight loss steps. In the initial stage, a slight weight loss between 25 °C to 100 °C was observed which corresponds to the moisture loss for both the membranes^64^. The second stage (100 to 250 °C) weight loss was due to the decomposition of carboxylic groups and grafted PVA side chains on the surface of both the membranes^65^. As the temperature continued to rise, a weight loss in the range of 250 °C to 400 °C in both membranes occurred due to the decomposition of functional groups and PVA molecules^66,67^. Furthermore, the weight loss between 400 °C to 500 °C in both the membranes was attributed to the oxidation of MWCNTs. In addition, the TGA curve indicated the JP immobilized BP/PVA membrane decomposed at a faster rate as compared to BP/PVA membrane between 250 °C to 350 °C. The steep and steady weight loss of JP immobilized BP/PVA membrane confirms enhanced thermal stability due to its highly-organized structure. The observed flat profiles at a temperature above 500 °C indicated that both metal catalyst and support remained as a residue after their onset temperature as they were not volatile. At this stage, the remaining weight of JP-immobilized BP/PVA was higher than that of BP/PVA membrane. Therefore, it can be inferred that the JP-immobilized BP/PVA membrane had a higher JP enzyme weight fraction of approximately 2.5 wt%, as calculated from TGA results.

## Conclusion

The biocompatible surface-modified BP/PVA nanocomposite membrane was synthesized and successfully used as support material for immobilization of jicama peroxidase. RSM is a useful tool to optimize the operating parameters of immobilization efficiency of the enzyme on BP/PVA membrane. A maximum enzyme immobilization efficiency of 81.74% was achieved at pH 6, 0.13 U/mL initial enzyme loading and 130 min immobilization time. BP/PVA film exhibits high enzyme loading capacity of 217 mg/g, owing to its highly porous structure, large surface-to-volume ratio, as well as strong electrostatic interactions between JP and BP/PVA. Various characterizations such as SEM, EDX, FTIR and TGA were performed and all confirmed the successful immobilization of JP on BP/PVA membrane. Immobilized JP showed significantly improved pH, thermal and storage stabilities in comparison with its free counterpart. Thus, the JP-immobilized BP/PVA nanocomposite membrane is a promising and potential nano-biocatalyst for practical applications in industry. These findings could further contribute to the construction of immobilized enzymes at a larger scale for industrial applications. The combination of immobilized enzymatic and nano-membrane technology is envisioned to bring about significant impacts of advanced enzyme immobilization technologies in various fields.

## Materials and Reagents

MWCNTs with a diameter ranging between 16–23 nm and 98% purity were obtained from previous study^68^. Jicama skin peels were supplied by local vendors in Miri, Sarawak. Poly (vinyl alcohol) (molecular weight, M_w_ = 31,000–50,000, 98–99% hydrolyzed), sulfuric acid (H_2_SO_4_), hydrochloric acid (HCl), hydrogen peroxide (H_2_O_2_), bovine serum albumin (BSA), sodium dihydrogen phosphate monohydrate, di-sodium hydrogen phosphate, boric acid, citric acid, and glutaraldehyde (C_5_H_8_O_2_) were purchased from Merck Eurolab GmBh Darmstadt, Germany. Nitric acid (HNO_3_), phenol detached crystal and absolute ethanol were purchased from Fisher Scientific, Loughborough, UK. 4-aminoantipyrene (4-AAP) was supplied by Acros Organic, New Jersey, USA. Distilled water was used all throughout the experiment. Chemicals of analytical grade were used in this study.

## Methods

### Synthesis of functionalized Buckypaper/PVA

Initially, surface modified MWCNTs was prepared by acid functionalization, as described in our previous work^67^. The surface modified MWCNTs was dispersed into 50 mL of ethanol for 2 min using ultrasonication. Then, the supernatant was filtered through a PTFE filter membrane under vacuum. After the formation of BP membrane, 2 wt% of PVA solution was poured onto its surface and filtered under vacuum. Finally, the BP/PVA membrane was dried in an oven for 10 min at 100 °C and then peeled off from the underlying PTFE membrane carefully.

### Extraction of Jicama Peroxidase

The extraction of peroxidases from jicama skin peels was performed by following the procedures of optimum extraction conditions reported by Chiong *et al*.^69^. Prior to the extraction process, the skin peels were washed with distilled water and air dried. Later, the skins were chopped coarsely and mixed with 0.1 M phosphate buffer with the ratio of 1:2 (plant to buffer ratio w/v) at pH 7. Next, the mixture was blended for 2 min and followed by constant stirring of the extract for 30 minutes. The extract was then filtered through four layers of cheesecloth and subject to centrifugation at 4,000 rpm for 20 min at 4 °C. The supernatant was sonicated for 1 minute before stored in 4 °C until further use.

### Immobilization of Enzyme on Buckypaper/PVA

The BP/PVA membranes were functionalized with glutaraldehyde (2.5% v/v) in phosphate buffer solution (PBS, 0.1 M, pH 7.0) for 60 min. Then, the BP/PVA membrane was washed with PBS (0.1 M, pH 7.0) to remove any remaining traces glutaraldehyde. The membranes were then immersed in peroxidase solution at different pH and enzyme loadings for several hours based on the experimental design obtained from Design Expert software. The membranes were under constant shaking of 150 rpm at room temperature throughout the enzyme immobilization process. After the process was completed, the unbound enzymes were then removed by rinsing with PBS (0.1 M, pH 7.0) for three times. The JP-immobilized BP membrane was left air dry and stored in 4 °C for further use.

### Enzyme Activity of Free and Immobilized Enzyme

Enzyme activities of JP were evaluated by measuring the absorbance of the colorimetric assay using a UV-Vis spectrophotometer. (Lambda 25 UV/Vis Double Beam, Perkin Elmer). The assay was prepared by following the optimum JP activity conditions reported by Wu, *et al*.^70^. The assay mixture consists of 500 µL of 0.1 M phosphate buffer pH 6, 250 µL of 9.6 mM 4-aminoantipyrene (4-AAP), 100 µL of 0.1 M phenol, and 100 µL of 2 mM hydrogen peroxide (H_2_O_2_). Finally, 50 µL of the free enzyme extract or 1 cm^2^ of the enzyme-immobilized membrane were added to the assay mixture. The absorbance of the assay was measured once per minute. One unit of enzyme activity was defined as the conversion of 1.0 µmol of H_2_O_2_ per minute at pH 6 and 25 °C. The enzyme activity was determined by using Eq. 2. All the experiments data were performed at least three times, and take the average of the results, with a standard deviation of less than 2%.

2 $$Enzyme\,\,activity\,(\frac{U}{m{L}_{enzyme}})=\frac{{\rm{\Delta }}{A}_{510nm}{V}_{assay}\,}{{V}_{enzyme}{\varepsilon }_{510nm}}$$

where ∆A_510_ nm represents the slope of absorbance versus time (min^−1^); V_assay_ and V_enzyme_ are the volume of the assay (µL) and volume of the enzyme (mL) respectively; ε_510_ nm is the extinction coefficient = 7100 M^−1^ cm^−1^

The immobilization efficiency (*IE*) can be determined by using Eq. 3. Besides, relative activity (*RA*) is expressed as the ratio of the enzyme activities to the maximum enzyme activities, shown as Eq. 4. The maximum enzyme activities for both free and immobilized enzyme were regarded as 100% of the enzyme activities to be analysed respectively.

3 $$IE\,( \% )=\frac{Total\,protein\,concentration\,of\,immobilized\,enzyme\,}{Total\,protein\,concentration\,of\,\,oaded\,enzyme}\times \,100$$

4 $$RA\,( \% )=\frac{Enzyme\,Activity\,}{Maximum\,Enzyme\,Activiity\,}\times 100$$

### Protein Concentration of Free and Immobilized Enzyme

The protein concentration of JP extracts was determined according to the standard assay method described by Bradford^71^. The calibration curve was constructed by using bovine serum albumin (BSA) as a standard. The absorbance was monitored at 595 nm by using UV-Vis spectrophotometer.

As for the immobilized enzyme, the amount of protein loaded on the membrane support was evaluated by the difference in the initial and final protein concentration, as well as the washings in the enzyme solutions. The enzyme loading, *P*_*i*_, was defined as the amount of enzyme immobilized per gram of the BP membrane, which is presented in Eq. 5.

5 $${P}_{i}(\frac{mg}{g})=\frac{({C}_{i}-{C}_{f})V-{C}_{w}{V}_{w}}{m}$$

where *C*_*i*_ and *C*_*f*_ are the initial and final concentrations of protein (mg/mL) respectively; *V* is the volume of total solution (mL); *V*_*w*_ is the volume of washing (mL), and *Cw* is the protein concentration in the washings (mg/mL); m is the dry weight of the membrane (g).

### Optimization of Immobilization Efficiency by Design Expert

In this study, Design Expert software (version 11.0, Stat-Ease Inc., Minneapolis, USA) was used to perform experimental design for analysis. To study the optimum operating parameters for peroxidase immobilization efficiency, RSM was used through the development of an appropriate model. RSM can also analyze and study the mechanism and the factors which influence the process.

FCCCD method was chosen for the RSM in the experimental design, in order to develop a quadratic model. The design output consisted of 16 sets of experimental runs, with 2 center points. The three process variables chosen were pH, initial loading of the enzyme, and immobilization time. The immobilization efficiency of the enzyme was defined as the response for this study. Factors were analyzed at high, center and low levels as shown in Table 3.

**Table 3:** Codes, ranges and levels of independent variables for the optimization of enzyme immobilization efficiency.

Variable	Factors	Units	Low (−1)	Center (0)	High (+1)
A	pH	—	4	6	8
B	Initial loading of enzyme	U/mL	0.1	0.5	0.9
C	Immobilization time	min	30	135	240

The quadratic polynomial equation was chosen for predicting the optimal points and is expressed in Eq. 6.

6 $$Y={\beta }_{0}+{\beta }_{1}{A}_{1}+{\beta }_{2}{B}_{2}+{\beta }_{3}{C}_{3}+{\beta }_{11}{A}^{2}+{\beta }_{22}{B}^{2}+{\beta }_{33}{C}^{2}+{\beta }_{12}AB+{\beta }_{13}AC+{\beta }_{23}BC$$

where Y represents the predicted response value (Immobilization efficiency), *β*_0_ is the offset term, *β*_1_ and *β*_2_ are linear coefficients, *β*_11_, *β*_22_ and *β*_33_ are quadratic coefficients. A, B and C are the independent variables which influence the response variable Y. The coefficients were estimated by performing 16 trials and the generated second order polynomial model was then validated by performing the experiment at given optimal conditions. The resulting model was examined by using ANOVA The validity of the model was determined based on Fischer’s F-test (F-values), associated probability (p-values), as well as regression coefficient values and lack of fit test. Finally, the mathematical model developed by RSM was validated by performing experiments.

### Effect of pH, temperature on Free and Immobilized Enzymes

The effects of pH on the relative activities of free and immobilized enzymes were evaluated by incubating the enzymes in 0.1 M phosphate buffer solution with various pH (ranging from pH 2 to pH 11) at room temperature. 1 M HCl and 1 M NaOH were prepared and used to adjust the pH of the enzymes. The enzyme activity was assayed as described above.

On the other hand, the effects of temperatures on the relative activities of both enzymes were determined by incubating the enzymes in phosphate buffer solution (0.1 M, pH 7) with various temperature ranging from 20 °C to 60 °C. The relative activity of the enzymes was assayed as above.

### Thermal and Storage Stabilities of Free and Immobilized Enzymes

As for thermal stability experiments, free and immobilized enzymes were incubated in phosphate buffer solution (0.1 M, pH 7) at 50 °C for 90 min of incubation. Aliquots of the samples were collected at every 15 min interval and the enzyme activities were measured under standard assay condition. For storage stabilities experiments, free and immobilized enzymes were incubated in phosphate buffer solution (0.1 M, pH 7) at 4 °C. The enzyme activities were measured at certain time intervals for 5 weeks.

### Characterization of BP and JP-Immobilized BP

In this study, several characterization studies of both BP membrane and enzyme immobilized BP membranes were conducted. Field Emission Scanning Electron Microscope (FEI Quanta 400 SEM) coupled with Energy Dispersive X-ray spectroscopy (EDX) was used to analyze the surface morphologies and elemental compositions of the membrane. Additionally, Fourier Transform Infrared (FTIR) spectroscopy (PerkinElmer FTIR) was used to analyze the functional groups of the membranes. Lastly, thermogravimetric analysis (TGA) (Mettler Toledo) was conducted to investigate the thermal stability, as well as to analyze the degradation of individual compositions of the samples. TGA analysis was carried out from 25 °C to 900 °C at a rate of 10 °C/min under nitrogen gas flow of 100 mL/min.
